# Metagenomic Diagnosis of Bacterial Infections

**DOI:** 10.3201/eid1411.080589

**Published:** 2008-11

**Authors:** Shota Nakamura, Norihiro Maeda, Ionut Mihai Miron, Myonsun Yoh, Kaori Izutsu, Chidoh Kataoka, Takeshi Honda, Teruo Yasunaga, Takaaki Nakaya, Jun Kawai, Yoshihide Hayashizaki, Toshihiro Horii, Tetsuya Iida

**Affiliations:** Osaka University, Suita, Japan (S. Nakamura, I.M. Miron, M. Yoh, K. Izutsu, C. Kataoka, T. Honda, T. Yasunaga, T. Nakaya, T. Horii, T. Iida); RIKEN Yokohama Institute, Yokohama, Japan (N. Maeda, J. Kawai, Y. Hayashizaki)

**Keywords:** high-throughput DNA sequencing, bacterial infections, acute diarrhea, detection, identification, diagnosis, bioterrorism, dispatch

## Abstract

To test the ability of high-throughput DNA sequencing to detect bacterial pathogens, we used it on DNA from a patient’s feces during and after diarrheal illness. Sequences showing best matches for *Campylobacter jejuni* were detected only in the illness sample. Various bacteria may be detectable with this metagenomic approach.

Infectious diseases are caused by various pathogens, including as-yet unidentified microorganisms. Because procedures for detecting and identifying pathogens vary according to the target microorganism, clinical examinations require a variety of media, reagents, and culture methods. In addition, conventional examination protocols usually require much labor, time, and skill, thus forming an obstacle to a prompt diagnosis.

Newly developed, “next-generation” DNA sequencers can determine >100 megabases of DNA sequences per run (*1*). These new technologies eliminate the bacterial cloning step used in traditional Sanger sequencing; instead, they amplify single isolated DNA molecules and analyze them with massively parallel processing. To develop a new system to promptly detect and identify various infectious pathogens, we tapped into the potential of these novel sequencers. We directly detected the causative pathogenic microbe in a clinical human sample (diarrheic feces) by means of unbiased high-throughput DNA sequencing.

## The Study

A 34-year-old man had become ill after eating dinner out with his family. After 3 days, severe diarrhea, stomach ache, and shivering developed in the only 3 persons (the patient plus 2 family members) who had eaten undercooked chicken that night. Four days after onset of clinical signs, feces were collected from the patient and stored in a freezer at –80°C. At a clinical laboratory in Osaka, Japan, conventional culture methods were used to examine the sample for possible bacterial enteropathogens (*2*), and specific reverse transcriptase–PCR was used to test for norovirus (*3*); however, no candidate pathogens were detected.

We therefore analyzed this fecal sample for possible pathogens by means of high-throughput DNA sequencing. DNA was extracted from the diarrhea sample (hereafter referred to as the illness DNA sample) with a QIAamp DNA Stool Mini Kit (QIAGEN, Valencia, CA, USA). After the man had completely recovered 3 months later, another fecal sample was collected (hereafter referred to as the recovery DNA sample) and maintained at –80°C until DNA extraction. Both DNA samples were subjected to unbiased high-throughput DNA sequencing with a GS20 sequencer (454 Life Sciences, Branford, CT, USA) (*4*).

Sequencing produced 96,941 effective sequences for the illness DNA sample and 106,327 for the recovery sample. The average length of the sequences was 102.1 bp. The DNA sequences obtained were searched with the BLASTN program for the National Center for Biotechnology Information nucleotide sequence database (http://blast.ncbi.nlm.nih.gov). The BLASTN output was then analyzed by using a classification system consisting of the Center’s taxonomy database and its searching system. This system, devised with the aid of Perl language (www.perl.com) and the MySQL database (www.mysql.com), facilitates the identification of scientific names and statistical analysis. The Figure shows the organisms from which the sequences in the database were derived that showed the best matches for the sequences queried (expect [E]-value <10^–5^). For both DNA samples, ≈20% of the total sequences showed the best matches for the currently reported bacterial DNA sequences. The Table shows the frequency distributions of species from which close matches for the sequences were derived (E-value <10^–40^). The most frequently detected bacterial species in both samples belonged to the phylum Bacteroidetes, the normal flora of the human intestine. No major differences were found in the frequency of the species between the illness and recovery DNA samples.

**Figure:**
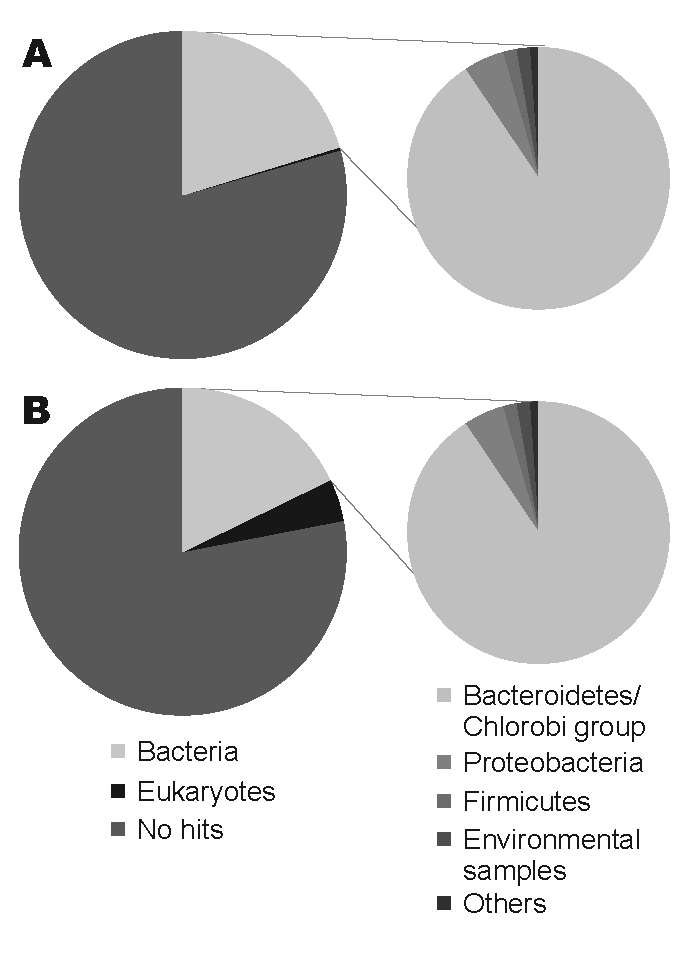
Comparison of the organisms from which the best matches for the sequences were derived from a BLASTN (http://blast.ncbi.nlm.nih.gov) search with an expect-value cutoff of 10^–5^. A) DNA from nondiarrheic fecal sample collected 3 months after patient had recovered. B) DNA from diarrheic fecal sample collected while patient was ill.

**Table:** Frequency distributions of species in fecal samples taken from patient during illness and after recovery, as determined by BLASTN*

Organism	No. (%)	
	Illness†	Recovery‡
*Bacteroides vulgatus*	5,944 (50.5)	4,743 (56.5)
*Homo sapiens*	2,955 (25.1)	84 (1.0)
*Parabacteroides distasonis*	818 (6.9)	1,283 (15.3)
*B. thetaiotaomicron*	767 (6.5)	1,046 (12.5)
*B. fragilis*	759 (6.4)	842 (10.0)
Uncultured bacterium	195 (1.7)	227 (2.7)
*Campylobacter jejuni*	156 (1.3)	0
*B. ovatus*	48 (0.4)	63 (0.8)
Uncultured *Bacteroides* spp.	20 (0.2)	19 (0.2)
*B. uniformis*	14 (0.1)	8 (0.1)

*BLASTN available from http://blast.ncbi.nlm.nih.gov. Expect-value cutoff 10^–40^. †Diarrheic fecal sample collected while patient was ill. Total sequences 96,941; total (100%) BLAST matches 11,777. ‡Nondiarrheic fecal sample collected 3 mo after patient had recovered. Total sequences 106,327; total (100%) BLAST matches 8,397.

A striking difference between the 2 samples, however, was that 156 sequences of the illness DNA sample showed best matches for the sequences derived from *Campylobacter jejuni*, but no sequences of the recovery DNA sample showed any such significant matches. The *C. jejuni* sequences from the illness DNA sample included many housekeeping genes, such as the genes for the ribosomal RNAs and DNA polymerases (Appendix Table); thus, they strongly suggested the presence of *C. jejuni* in the illness fecal sample.

Because *C. jejuni* is a bacterium that causes acute gastroenteritis and is normally not present in the intestines of healthy persons (*5*,*6*), these results prompted us to reexamine the illness fecal sample for *C. jejuni.* For the illness sample but not the recovery DNA sample, *Campylobacter*-specific PCR (*7*) produced a typical banding pattern that is unique to *C. jejuni* (data not shown). The recovery rate of *Campylobacter* spp. from patient specimens substantially decreases when the specimens are frozen before isolation (*8*). To obtain higher recovery of *Campylobacter* spp. and thus validate the presence of *C. jejuni* in the illness sample, we performed cultures with enrichment and selective media again on the frozen illness fecal sample (*5*). *C. jejuni–*like bacteria with corkscrew motility grew on selective agar plates. Biochemical identification using the API Campy kit (API-bioMérieux, Marcy L’Etoile, France) demonstrated that the organism was *C. jejuni,* thus proving its presence in the illness fecal sample.

## Conclusions

We directly detected a bacterial pathogen in a patient sample by using high-throughput DNA sequencing. This finding implies that basically any kind of bacterial pathogen may be detectable with a common procedure. The method is directly applicable not only to fecal samples but also to other types of clinical samples; it could detect and identify bacterial pathogens that are usually difficult to ascertain with conventional examination procedures. Because this novel approach can be expected to have major potential for detection of pathogens in various infectious diseases, it warrants further investigation.

The approach reported here also enabled us to directly analyze the ratio of pathogenic to commensal bacteria in the human intestine. Assessment of the relative population of intestinal bacteria would enable us to investigate the dynamics of bacterial pathogens in human intestines, in relation to associated intestinal microbial flora, during infectious disease processes.

Many causative agents of emerging infectious diseases are of animal origin, and many are previously identified microbes (*9*,*10*). Because a vast amount of genome information about various microorganisms is continually being accumulated in databases, the approach we used will become increasingly useful. Recent metagenomic studies have identified unknown virus pathogens (*11*–*13*). Using the present approach to analyze various clinical cases, especially of outbreaks of infectious diseases with as-yet unidentified causative agents, may lead to the discovery of novel bacteria that are currently not known to be pathogenic to humans.

The current cost for high-throughput sequencing may limit the use of this method to specialized purposes, such as the hunt for novel pathogens for research or detection of bioterrorism (*14*). However, because the progress of DNA sequencing technology has been rapid (*1*), the cost, time, and labor for sequencing have been greatly reduced, and this trend will likely continue for the foreseeable future (*15*). Therefore, high-throughput DNA sequencing may soon be adopted as the main method for examining microorganisms in major clinical laboratories. The data presented here represent an example of this major innovation in the field of clinical examination for causative agents of infectious diseases.

## Supplementary Material

Appendix Table BLASTN matches for Campylobacter jejuni sequences*
