# Supplementary material for: Metagenomic Diagnosis of Bacterial Infections
Source: Emerg Infect Dis. 2008 Nov;14(11):1784–6. doi: 10.3201/eid1411.080589 (PMC2630750; doi:10.3201/eid1411.080589)
Supplement: Appendix Table — BLASTN matches for Campylobacter jejuni sequences* [file 08-0589_appT-s1.pdf]

Appendix Table. BLASTN matches for *Campylobacter jejuni* sequences*

Accession no.	Start	End	E-value†	BLAST hit
CP000538	84217	84073	6.00E-75	Flagellar motor switch protein (*fliY*)
CP000538	820953	821083	1.00E-66	Ribosomal protein S15 (*rpsO*)
AF130466	7941	7806	2.00E-65	Putative glycosyltransferase
AF130466	7941	7806	2.00E-65	Putative glycosyltransferase
CP000538	896576	896450	3.00E-64	Peptidyl-arginine deiminase family protein
CP000538	248199	248324	1.00E-63	Biotin sulfoxide reductase, molybdopterin containing oxidoreductase
CP000538	222134	222259	1.00E-63	Conserved hypothetical protein
AF167344	22157	22033	4.00E-63	Heavy metal translocating P-type ATPase
CP000538	1498737	1498860	2.00E-62	Translation initiation factor IF-1 (*infA*)
CP000538	513409	513287	6.00E-62	Conserved hypothetical protein
CP000538	864240	864119	2.00E-61	GTP-binding protein YchF
CP000538	185407	185288	4.00E-60	Transporter
CP000538	113295	113413	1.00E-59	Hypothetical protein
CP000538	461714	461596	1.00E-59	Putative periplasmic protein
CP000538	556839	556714	2.00E-59	MOSC domain protein
AL111168	1233367	1233484	6.00E-59	3-oxoacyl-(acyl-carrier-protein) synthase
CP000538	774644	774761	6.00E-59	tRNA pseudouridine synthase A (*truA*)
CP000538	774644	774761	6.00E-59	tRNA pseudouridine synthase A (*truA*)
CP000768	196036	196153	6.00E-59	Putative periplasmic protein
DQ493919	2207	2083	6.00E-59	Heat-inducible transcription repressor (*hrcA*)
CP000025	212978	213094	2.00E-58	Hypothetical protein
CP000538	1310351	1310467	2.00E-58	Catalase (*katA*)
CP000768	196036	196152	2.00E-58	Putative periplasmic protein

CP000538	1521206	1521082	3.00E-58	Hemin ABC transporter
CP000538	339049	338934	9.00E-58	RND efflux system, inner membrane transporter (*cmeB*)
CP000538	809625	809502	9.00E-58	Site-specific recombinase, phage integrase family
CP000538	461702	461588	3.00E-57	Putative periplasmic protein
CP000538	1522395	1522509	3.00E-57	Putative radical SAM domain protein
CP000538	451930	452044	3.00E-57	Translation elongation factor G (*fusA*)
CP000538	774644	774758	3.00E-57	tRNA pseudouridine synthase A (*truA*)
CP000025	211254	211367	1.00E-56	Tail tape measure protein, TP901 family
CP000538	303524	303637	1.00E-56	3-oxoacyl-(acyl-carrier-protein) synthase III (*fabH*)
CP000538	1471284	1471397	1.00E-56	NADH-quinone oxidoreductase, N subunit (*nuoN*)
CP000538	789384	789271	1.00E-56	Putative lipoprotein
AY102622	19934	19822	5.00E-56	Motility accessory factor
AY102622	19934	19822	5.00E-56	Motility accessory factor
CP000538	1371661	1371549	5.00E-56	Peptide chain release factor 2 (*prfB*)
DQ174139	1132	1020	5.00E-56	16S ribosomal RNA (*rrsA*)
CP000538	39117	39006	2.00E-55	Intergenic sequence
CP000538	236407	236518	2.00E-55	Major facilitator superfamily protein
AL111168	905046	905156	8.00E-55	Putative periplasmic protein
AL111168	905046	905156	8.00E-55	Putative periplasmic protein
BX545860	5780	5890	8.00E-55	Putative methyltransferase
CP000538	346235	346125	8.00E-55	Conserved hypothetical protein
CP000538	346235	346125	8.00E-55	Conserved hypothetical protein
CP000768	196036	196153	8.00E-55	Putative periplasmic protein
AL111168	1123274	1123154	3.00E-54	Phosphate transporter family protein
CP000025	211681	211572	3.00E-54	Tail tape measure protein, TP901 family
CP000538	1126394	1126503	3.00E-54	Putative dihydroorotase
CP000538	461697	461588	3.00E-54	Putative periplasmic protein
CP000538	185853	185962	3.00E-54	Putative transporter
AY102622	29478	29354	1.00E-53	Motility accessory factor

CP000538	727769	727649	1.00E-53	Conserved hypothetical protein
CP000538	1265191	1265083	1.00E-53	Hypothetical protein
AL111168	756034	755923	5.00E-53	Lipid export ABC transport protein (*msbA*)
CP000538	267947	268054	5.00E-53	Chemotaxis protein CheA (*cheA*)
CP000538	339049	338934	5.00E-53	RND efflux system, inner membrane transporter (*cmeB*)
CP000538	339049	338934	5.00E-53	RND efflux system, inner membrane transporter (*cmeB*)
AF387299	7	127	2.00E-52	Putative integral membrane protein
AL111168	14747	14853	2.00E-52	Glutamate synthase small subunit (*gltD*)
AL111168	145443	145330	2.00E-52	Putative ABC transporter
CP000538	1498249	1498127	2.00E-52	Conserved domain protein
CP000538	531483	531377	2.00E-52	GatB/Yqey family protein
CP000538	185157	185263	2.00E-52	Transporter
CP000538	841649	841754	7.00E-52	Amino acid ABC tansporter
CP000538	419915	419806	7.00E-52	DNA polymerase III subunit epsilon
CP000538	667737	667842	7.00E-52	DNA polymerase III, alpha subunit (*dnaE*)
CP000538	933041	932936	7.00E-52	Porphobilinogen synthase (*hemB*)
CP000768	1832415	1832520	7.00E-52	3-isopropylmalate dehydratase, large subunit (*leuC*)
CP000549	41769	41881	8.00E-52	Tetracycline resistance gene (*tetO*)
CP000538	1263606	1263735	9.00E-52	1-deoxy-D-xylulose 5-phosphate reductoisomerase (*dxr*)
CP000538	1190525	1190405	9.00E-52	Low molecular weight phosphotyrosine protein phosphatase family
CP000538	1204980	1205109	9.00E-52	Tyrosyl-tRNA synthetase (*tyrS*)
CP000538	675765	675661	3.00E-51	Molybdenum cofactor biosynthesis protein (*mog*)
CP000538	84217	84089	4.00E-51	Flagellar motor switch protein (*fliY*)
AY330117	206	309	1.00E-50	DNA gyrase subunit B (*gyrB*)
CP000538	123002	123105	1.00E-50	ATP synthase F1, alpha subunit (*atpA*)
CP000538	346235	346125	1.00E-50	Conserved hypothetical protein
CP000538	346235	346125	1.00E-50	Conserved hypothetical protein
CP000538	346235	346125	1.00E-50	Conserved hypothetical protein
CP000538	7914	7811	1.00E-50	Na+/H+ antiporter family protein

CP000538	774644	774758	1.00E-50	tRNA pseudouridine synthase A (*truA*)
CP000538	44292	44394	4.00E-50	23S ribosomal RNA AL111168 (*rrlA*)
CP000538	385697	385595	4.00E-50	Putative GMC oxidoreductase subunit
CP000538	385697	385595	4.00E-50	Putative GMC oxidoreductase subunit
CP000025	183185	183055	2.00E-49	Conserved hypothetical protein
CP000538	126825	126716	2.00E-49	MotA/TolQ/ExbB proton channel family protein
CP000538	802224	802325	2.00E-49	Signal peptidase I (*lepB*)
CP000538	554078	553978	6.00E-49	Chemotaxis protein MotB, putative
CP000538	554078	553978	6.00E-49	Putative chemotaxis protein MotB
CP000538	1413419	1413519	6.00E-49	Sodium/proline permease (*putP*)
CP000538	410969	411069	6.00E-49	TenA/Thi-4 family protein
CP000538	24172	24065	7.00E-49	Methyl-accepting chemotaxis protein
CP000538	24172	24065	7.00E-49	Methyl-accepting chemotaxis protein
AL111168	1335881	1335995	8.00E-49	Putative triosephosphate isomerase (*tpiA*)
CP000538	1140999	1141123	8.00E-49	DedA family protein
CP000538	926620	926742	8.00E-49	Membrane protein
CP000538	579903	580018	9.00E-49	[NiFe] hydrogenase maturation protein (*hypF*)
AL111168	1337472	1337373	3.00E-48	Glyceraldehyde 3-phosphate dehydrogenase (*gapA*)
CP000538	530419	530539	3.00E-48	3,4-dihydroxy-2-butanone 4-phosphate synthase (*ribB*)
CP000538	530419	530539	3.00E-48	3,4-dihydroxy-2-butanone 4-phosphate synthase (*ribB*)
CP000538	355517	355616	3.00E-48	GTPase family protein
CP000538	24172	24065	3.00E-48	Methyl-accepting chemotaxis protein
CP000538	1264854	1264755	3.00E-48	Phosphatidate cytidylyltransferase (*cdsA*)
CP000538	1277793	1277892	3.00E-48	Polyphosphate kinase (*ppk*)
CP000538	1188103	1187981	3.00E-48	Putative isomerase
CP000538	1190500	1190401	4.00E-48	Low molecular weight phosphotyrosine protein phosphatase family
BX545860	7068	7185	1.00E-47	Putative methyltransferase
CP000025	1648716	1648818	1.00E-47	NADH dehydrogenase I chain D (*nuoD*)
CP000538	27981	28104	1.00E-47	RNA pseudouridine synthase family protein

CP000538	27981	28104	1.00E-47	RNA pseudouridine synthase family protein
CP000538	1014735	1014833	2.00E-47	Uroporphyrinogen-III synthase (*hemD*)
CP000538	448381	448284	4.00E-47	DNA-directed RNA polymerase, beta' subunit (*rpoC*)
CP000538	24172	24065	4.00E-47	Methyl-accepting chemotaxis protein
CP000768	1780736	1780635	4.00E-47	Conserved hypothetical protein
CP000768	1780736	1780635	4.00E-47	Conserved hypothetical protein
CP000768	1780736	1780635	4.00E-47	Conserved hypothetical protein
AF167344	1857	1761	1.00E-46	Putative UDP-glucose-4-epimerase
CP000538	922881	922977	1.00E-46	CjaA protein (*cjaA*)
CP000538	1114408	1114504	1.00E-46	Ubiquinol--cytochrome c reductase, cytochrome b subunit (*petB*)
AL111168	1335439	1335343	2.00E-46	Enoyl- [acyl-carrier-protein] reductase (*fabI*)
CP000538	257912	257804	2.00E-46	ATP-dependent Clp protease, ATP-binding subunit (*clpX*)
CP000538	1146400	1146304	2.00E-46	Peptidase, M23/M37 family
CP000538	402908	402798	2.00E-46	Phospho-N-acetylmuramoyl-pentapeptide–transferase (*mraY*)
CP000538	409993	410093	2.00E-46	Succinate dehydrogenase, C subunit (*sdhC*)
CP000538	84217	84121	3.00E-46	Flagellar motor switch protein (*fliY*)
CP000538	1528838	1528743	6.00E-46	L-serine ammonia-lyase (*sdaA*)
DQ174139	314	219	6.00E-46	16S ribosomal RNA (*rrsA*)
CP000538	1413419	1413522	7.00E-46	Sodium/proline permease (*putP*)
CP000768	176574	176681	7.00E-46	Iron ABC transporter
CP000025	211681	211572	3.00E-45	Tail tape measure protein, TP901 family
CP000538	770432	770331	3.00E-45	Phosphopantothenoylcysteine decarboxylase/phosphopantothenate--cysteine ligase (*coaBC*)
AL111168	831387	831480	9.00E-45	D-3-phosphoglycerate dehydrogenase (*serA*)
CP000538	4919	4822	9.00E-45	DNA gyrase, B subunit (*gyrB*)
CP000538	458602	458699	9.00E-45	Putative periplasmic protein
CP000538	822020	821927	1.00E-44	Cell division protein FtsK, putative
CP000538	1094669	1094778	1.00E-44	Copper-translocating P-type ATPase
CP000538	24172	24065	4.00E-44	Methyl-accepting chemotaxis protein
CP000538	530419	530539	5.00E-44	3,4-dihydroxy-2-butanone 4-phosphate synthase (*ribB*)

AY681275	1023	931	6.00E-44	Cytochrome c family protein
DQ140271	1883	1784	1.00E-43	UDP-GlcNAc/Glc 4-epimerase
CP000538	296556	296647	2.00E-43	1-deoxy-D-xylulose-5-phosphate synthase (*dxs*)
CP000538	554181	554272	2.00E-43	Chemotaxis protein MotB
AL111168	1133069	1132975	6.00E-43	5,10-methylenetetrahydrofolate reductase (*metF*)
CP000768	1780736	1780635	6.00E-43	Conserved hypothetical protein
CP000538	1028222	1028113	7.00E-43	Protein-export membrane protein SecF (*secF*)
CP000538	741897	741782	7.00E-43	Putative aminotransferase
CP000538	535230	535341	7.00E-43	S-adenosylmethionine:tRNA ribosyltransferase-isomerase(*queA*)
CP000538	535230	535341	7.00E-43	S-adenosylmethionine:tRNA ribosyltransferase-isomerase (*queA*)
CP000538	221318	221229	2.00E-42	Ribonucleoside-diphosphate reductase, beta subunit (*nrdB*)
CP000538	147831	147943	3.00E-42	Conserved hypothetical protein
CP000538	348795	348678	1.00E-41	ATPase, AAA family protein
CP000538	442651	442738	3.00E-41	DNA-directed RNA polymerase, beta subunit (*rpoB*)
CP000538	442651	442738	3.00E-41	DNA-directed RNA polymerase, beta subunit (*rpoB*)
AY681249	350	459	4.00E-41	Conserved hypothetical protein
DQ518908	8640	8529	4.00E-41	Putative UDP-N-acetylglucosamine 2-epimerase (*neuC1*)

*BLASTN available from http://blast.ncbi.nlm.nih.gov. MOSC, molybdenum cofactor sulfurase C; ABC, ATP binding cassette; SAM, S-adenosylmethinione; GMC, glucose–methanol–choline.

†Expect (E)–value cutoff 10^{-40}.
